# Expression Profile of Drug and Nutrient Absorption Related Genes in Madin-Darby Canine Kidney (MDCK) Cells Grown under Differentiation Conditions

**DOI:** 10.3390/pharmaceutics4020314

**Published:** 2012-06-18

**Authors:** Yong Quan, Yisheng Jin, Teresa N. Faria, Charles A. Tilford, Aiqing He, Doris A. Wall, Ronald L. Smith, Balvinder S. Vig

**Affiliations:** 1 Drug Product Science and Technology; Bristol-Myers Squibb Company, 1 Squibb Drive, New Brunswick, NJ 08903, USA; Email: yong.quan@bms.com (Y.Q.); teresa.n.faria@gmail.com (T.N.F.); doriswg@comcast.net (D.A.W.); ronald_smithl@merck.com (R.L.S.); 2 Applied Genomics; Bristol-Myers Squibb Company, 311 Pennington-Rocky Hill Road, Hopewell, NJ 08534, USA; Email: dilbert@gmail.com (Y.J.); charles.tilford@bms.com (C.A.T.); aiqing.he@bms.com (A.H.)

**Keywords:** MDCK, microarray, Transwell^®^ membranes, transporters, CYP enzymes

## Abstract

The expression levels of genes involved in drug and nutrient absorption were evaluated in the Madin-Darby Canine Kidney (MDCK) *in vitro* drug absorption model. MDCK cells were grown on plastic surfaces (for 3 days) or on Transwell^®^ membranes (for 3, 5, 7, and 9 days). The expression profile of genes including ABC transporters, SLC transporters, and cytochrome P450 (CYP) enzymes was determined using the Affymetrix^®^ Canine GeneChip^®^. Expression of genes whose probe sets passed a stringent confirmation process was examined. Expression of a few transporter (*MDR1*, *PEPT1* and *PEPT2*) genes in MDCK cells was confirmed by RT-PCR. The overall gene expression profile was strongly influenced by the type of support the cells were grown on. After 3 days of growth, expression of 28% of the genes was statistically different (1.5-fold cutoff, *p* < 0.05) between the cells grown on plastic and Transwell^®^ membranes. When cells were differentiated on Transwell^®^ membranes, large changes in gene expression profile were observed during the early stages, which then stabilized after 5–7 days. Only a small number of genes encoding drug absorption related SLC, ABC, and CYP were detected in MDCK cells, and most of them exhibited low hybridization signals. Results from this study provide valuable reference information on endogenous gene expression in MDCK cells that could assist in design of drug-transporter and/or drug-enzyme interaction studies, and help interpret the contributions of various transporters and metabolic enzymes in studies with MDCK cells.

## 1. Introduction

Cell-based *in vitro* drug permeation models are very attractive because they are amenable to high-throughput screening and can be implemented at a relatively low cost compared to the *in vivo* models [1]. Caco-2 *in vitro* cell culture model is a well-established and widely used model to characterize the human intestinal permeability of compounds. Studies have shown that Caco-2 permeability of the compounds that are absorbed passively correlated with their human intestinal permeability; however, this is not the case for compounds that depend on membrane transporters for absorption across intestinal barriers [2]. For example, β-lactam antibiotics and angiotensin-converting enzyme (ACE) inhibitors are well-absorbed *in vivo*, but not in the Caco-2 model, presumably due to lower expression of the transporters, such as PepT1 in Caco-2 cells than in human small intestine [1,2]. In addition, Caco-2 cells generally require a 2–3 week growth period before they fully differentiate on Transwell^®^ membranes [3]. To overcome the issues mentioned above, some investigators have suggested using alternative *in vitro* cell culture models [1,4,5]. 

Madin-Darby Canine Kidney (MDCK) cell line, originally derived from dog kidney epithelial cells, is one such alternative *in vitro* cell culture model [6]. MDCK cells, like Caco-2 cells, form polarized monolayers with tight junctions, in much shorter culture time [5,7]. MDCK cells grown on the polycarbonate or nitrocellulose Transwell^®^ membranes, differentiate into fully polarized columnar cells with an apical membrane containing microvilli and a basolateral membrane where cells attach to each other and to the Transwell^®^ membranes [8]. MDCK cells grown on non-permeable supports, such as plastic tissue culture plates, do not fully polarize [8]. For example, when only apical surface of MDCK cells is exposed to culture media, nutrient absorption systems such as methionine and transferrin uptake proteins are expressed on the apical surface, while they are expressed on the basolateral surface *in vivo* and in MDCK cells grown on the Transwell^®^ membranes [8,9,10]. 

MDCK cells have been widely used to study tight-junction formation and establishment of epithelial cell polarity [11,12]. More recently, they have also been used as an *in vitro* cell culture model to predict human intestinal permeability of drug candidates and to conduct drug-transport studies [13]. The permeation values of the passively absorbed compounds in MDCK cells correlated well with those in Caco-2 cells [13], making MDCK cells a very attractive permeability screening tool for such compounds. Further, many MDCK cell lines over-expressing membrane transporters of interest have been generated to study the effect of individual transporters on drug absorption and disposition [4,14,15,16,17,18,19,20,21]. Many drugs are substrates of multiple transporters and CYP metabolic enzymes, for example, mitoxantrone, bisantrene, topotecan, prazosin, and rhodamine 123 can be recognized by both ABCB1 (MDR1) and ABCG2 (BCRP), while daunorubicin, doxorubicin and epirubicin can be recognized by ABCB1 (MDR1/PGP), ABCG2 (BCRP), and ABCC1 (MRP1) [22]. Therefore, endogenous expression of membrane transporters and CYP enzymes in differentiated MDCK cells, could have significant impact on a compound’s absorption and complicate data interpretation. Knowledge of the endogenous expression of absorption related genes could be very useful to improve assessment of drug absorption in MDCK *in vitro* model. This information could also be helpful to design and interpret the drug-transporter and/or drug-enzyme interaction studies for the gene of interest in over-expressed systems. 

Although it has been suggested that MDCK cells have low endogenous expression of transporters and metabolic enzymes [5], a detailed and complete expression study for drug absorption related genes in this cell line is lacking [23]. Here we report results of a systematic study in which the global gene expression profiles of MDCK cells grown on Transwell^®^ membranes and on plastic surfaces were evaluated using an Affymetrix Canine GeneChip. We focused particularly on classes of genes involved in drug and nutrient absorption such as solute carrier transporters (SLC), efflux transporters (ABC), and phase I drug metabolism enzymes (CYP). 

## 2. Results

MDCK cells, when grown on the Transwell^®^ membranes, undergo differentiation as they reach confluency [24,25]. In the current study; MDCK cells, grown on the Transwell^®^ membranes, formed tight junctions and reached confluency by day 3. This was indicated by an increase in TEER value by day 3 that, after dropping slightly, became stable at approximately 200 Ω cm^2^ after day 5 (data not shown). MDCK cells grown on the Transwell^®^ membranes resulted in much denser monolayers than the cells grown on plastic plates for the same period (7 days). This probably occurs because fully differentiated cells grown on Transwell^®^ membranes elongate along the apical and basal axis [8], leaving more space for cell proliferation. To study the gene expression profile of MDCK cells during the differentiation process, MDCK cells grown on the Transwell^®^ membranes were harvested at various time points (differentiated for 3, 5, 7 and 9 days), including before and after TEER values were stabilized. To evaluate the difference in the gene expression profiles of MDCK cells grown on the Transwell^®^ membranes and plastic plates, MDCK cells grown on plastic plates for 3 days were harvested. For convenience, MDCK cells grown on plastic for 3 days were designated as 3d-P and MDCK cells differentiated on the Transwell^®^ membranes for 3, 5, 7, and 9 days were designated as 3d-T, 5d-T, 7d-T, and 9d-T, respectively. The nomenclature of ABC and SLC transporters recommended by the Human Genome Organization (HUGO) Nomenclature Committee was used in this paper, though some conventional names of the transporters were also used.

### 2.1. Overview of Expression Profiles under Differentiation Conditions

The Affymetrix Canine GeneChip used in this study contained 23,863 unique probe sets representing 16,145 full length genes. Application of a stringent confirmation process to the 23,863 probe sets (see Materials and Methods for details) resulted in 7908 confirmed probe sets representing 6339 unique genes. Since there are 25,599 genes assigned to dog genome in the LocusLink (Entrez), 6339 genes represent 24.8% of the dog genome (Table 1). However, these genes are well annotated with high level of confidence. 

**Figure 1:**
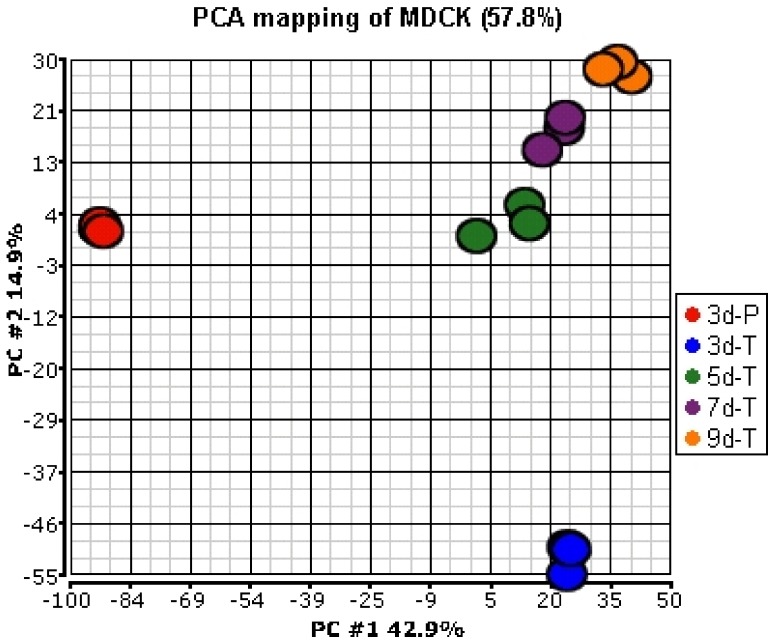
Principal Component Analysis (PCA) of gene expression profiles of MDCK cells in various culture conditions. Expression values from a total of 5554 confirmed probe sets that were considered present in at least one condition were used for analysis.

Out of the 7908 confirmed probe sets, 5554 were present in at least one of the growth conditions investigated here. Principal Component Analysis (PCA) was applied to the signals of 5554 confirmed and present probe sets, to compare global gene expression profiles between the MDCK cells grown on plastic and the Transwell^®^ membranes and among the cells grown for various culture times on the Transwell^®^ membranes. The results indicate that the expression profiles of the MDCK cells grown on plastic plates (3d-P) were very different from those grown on the Transwell^®^ membranes (Figure 1). Among the MDCK cells grown on the Transwell^®^ membranes, the expression profiles for the cells grown for 3 days were different from the cells grown for 5 days; fewer changes in the expression profiles were observed as the cells were allowed to differentiate for longer duration (5, 7, and 9 days). 

**Table 1:** Summary of probe sets in the Canine GeneChip used for analysis in this study.

	Number of probe sets	Number of valid probe sets	Number of unique genes	Number of genes signal detected	Number of genes signal not detected
Total	23,863 *	7908	6339	ND	ND
ABC	35	21	21	13	8
SLC	157	106	83	36	47
CYP	37	18	14	2	12

ND: not determined. * Total no. of unique gene is 16,145.

These results were confirmed by a pair-wise analysis of the gene expression data. Expression of 28.2% of the probe sets was statistically significantly different (≥1.5-fold) between the cells grown on plastic and the Transwell^®^ membranes (Table 2). In contrast, expression of only 4.2%, 0.4%, and 0.4% of the probe sets was statistically significantly different (≥1.5-fold) between the cells grown on the Transwell^®^ membranes for 3 and 5 days, 5 and 7 days, and 7 and 9 days, respectively. 

**Table 2:** Pair-wise analysis of gene expression profiles of MDCK cells grown under various culture conditions.

Culture condition comparison	No. (%) of probe sets that changed significantly *	Genes up-regulated	Genes down-regulated
3d-T *vs*. 3d-P	1565 (28.2%)	761	804
5d-T *vs*. 3d-T	232 (4.2%)	124	108
7d-T *vs*. 5d-T	22 (0.4%)	17	5
9d-T *vs*. 7d-T	22 (0.4%)	18	4

* Expression difference cut-off: ≥1.5-fold and *p* < 0.05.

### 2.2. Expression of Drug and Nutrient Absorption Related Genes during Cell Culture on the Transwell^®^ Membranes

Expression levels of the selected drug and nutrient absorption related genes as well as changes in their expression in MDCK cells grown on the Transwell^®^ membranes are discussed in detail below.

#### 2.2.1. ATP-Binding Cassette (ABC) Transporter Family Members Expression

In the Canine GeneChip, 35 probe sets represent 24 unique ABC transporter genes, 21 of which passed the confirmation process (Table 1). Out of the 21 confirmed ABC transporter genes, 13 were present in at least one of the growth conditions, while 8 were not detected. Expression of 8 and 2 ABC transporter genes was statistically different between 3d-T *vs*. 3d-P and 5d-T *vs*. 3d-T, respectively (Table 3). Expression of the ABC transporter genes did not differ significantly beyond day 5 (5d-T).

**Figure 2:**
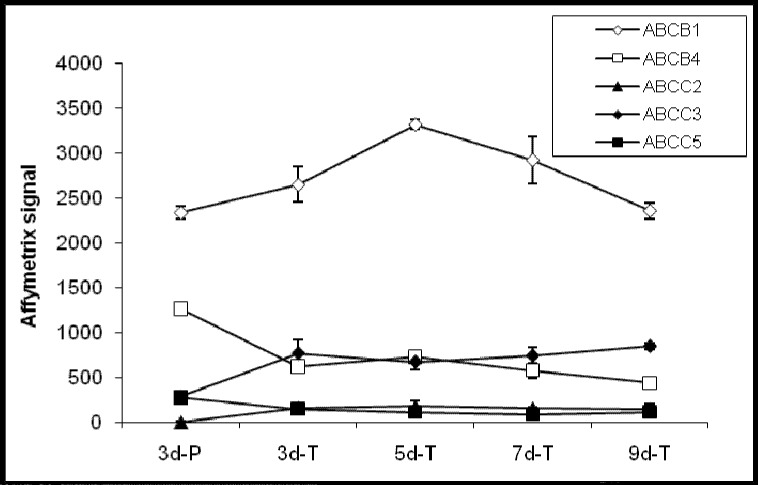
Expression profiles of ABC transporter genes involved in the drug absorption process.

Out of the 21 confirmed ABC transporter genes present in the Canine GeneChip, eight genes are involved in drug absorption. Of these, 5 genes; ABCB1 (MDR1), ABCB4 (MDR3), ABCC2 (MRP2), ABCC3 (MRP3), and ABCC5 (MRP5) were detected and 3; ABCB11 (BSEP), ABCC1 (MRP1), and ABCG2 (BCRP) were not detected in MDCK cells (Figure 2). A number of ABC transporters (ABCA5, ABCB2, ABCB3, ABCB6, ABCD3, ABCD4, ABCF1, and ABCF2), whose functions are not known at this time were also detected in MDCK cells. Among these, ABCA5 exhibited highest hybridization signal and most significant change in expression (4-fold) between the cells grown on Transwell^®^ membranes and plastic (Table 3). Additional ABC genes not detected in MDCK cells were ABCA6, ABCB9, ABCC11 (MRP8), ABCD2, and ABCF3. 

#### 2.2.2. Solute Carrier (SLC) Transporter Family Members Expression

In the Canine GeneChip, 157 probe sets represent SLC transporter genes, 106 of which passed the confirmation process (Table 1). Only 36 unique SLC genes were detected in MDCK cells (see supporting information for a detailed list of SLC transporters represented in Canine GeneChip). SLC genes whose expression differed significantly (1.5-fold cutoff) under the various growth conditions are summarized in Table 4. Similar to the ABC transporters, expression of the SLC transporters showed the most difference between 3d-T *vs*. 3d-P. Expression of 17, 6, 3 and 0 transporter genes showed statistically significant change among 3d-T *vs*. 3d-P, 5d-T *vs*. 3d-T, 7d-T *vs*. 5d-T and 9d-T *vs*. 7d-T, respectively (Table 4). 

**Table 3:** ABC transporters that exhibited statistically significant change in expression under various culture conditions.

Gene symbol	Synonym (RefSeq ID)	Function	3d-T/3d-P	5d-T/3d-T	7d-T/5d-T	9d-T/7d-T
ABCA5	(XM_537573)	Unknown	* 4.0	* −1.5	1.2	1.3
ABCB2	TAP1, RING4, ABC17, APT1, (XM_532101)	half-abc transporter, peptide transporter	* −2.3	* 1.6	1.1	1.2
ABCB3	TAP2, RING11, (XM_532099)	half-abc transporter, peptide transporter	* −1.7	1.2	1.1	−1.2
ABCB4	MDR-3, PGP3 (XM_539403)	biliary phosphatidylcholine secretion from hepatocytes	* −2.0	1.2	−1.3	−1.3
ABCC2	MRP2, CMOAT (NM_001003081)	efflux pump for drugs, anionic conjugates with glutathione, sulfate, or glucuronosyl	* Transwell^®^s only	1.1	−1.1	−1.1
ABCC3	MRP3, cMOAT2, MOAT-D (XM_548204)	transport of biliary and intestinal excretion of organic anions including bile salts	* 2.7	−1.1	1.1	1.1
ABCC5	MRP5 (XM_535820)	organic anion transporter for cyclic nucleotides and some nucleoside monophosphates	* −1.8	−1.3	−1.3	1.3
ABCD4	PXMP1L, P70R (XM_547903)	possible heterodimer for peroxisomal ABC transporter involved in import of fatty acids and/or fatty acyl-CoAs	* 2.4	−1.3	1.3	−1.3

* Indicates statistically significant change of 1.5-fold or higher; Negative ratios indicate decreased expression; Detected and statistically different (8): see table above; Detected but not statistically different (5): ABCB1 (MDR1), ABCB6, ABCD3, ABCF1, and ABCF2; Not detected (8): ABCA6, ABCB9, ABCB11, ABCC1 (MRP1), ABCC11, ABCD2, ABCF3, and ABCG2 (BCRP).

**Table 4:** SLC transporters that exhibited statistically significant change in expression under various culture conditions.

Gene symbol	Synonym (RefSeq ID)	Function	3d-T/3d-P	5d-T/3d-T	7d-T/5d-T	9d-T/7d-T
SLC1A1	EAA3, EAAT3, EAAC1 (NM_001003139)	high affinity glutamate and neutral amino acid transporter	* 7.0	* −1.7	−1.0	−1.1
SLC2A1	GLUT1 (XM_539554)	facilitative GLUT transporter	* −2.0	* 1.5	−1.1	1.1
SLC4A11	NaBC1, BTR1 (XM_542919)	bicarbonate transporter	* −2.2	* 1.6	−1.0	−1.1
SLC5A6	SMVT (XM_532905)	sodium glucose co-transporter	* −1.9	1.1	−1.1	−1.2
SLC6A12	BGT1 (NM_001003322)	sodium- and chloride- dependent neurotransmitter transporter	* Transwell^®^s only	1.3	* 1.8	1.4
SLC15A2	PEPT2 (XM_545128)	proton oligopeptide co-transporter	* Transwell^®^s only	* 1.5	* −1.5	1.1
SLC16A13	MCT13 (XM_844349)	monocarboxylate transporter	* 1.9	1.2	−1.2	1.1
SLC22A2	OCT2 gene (XM_533466)	organic cation transporter	* Transwell^®^s only	−1.2	1.1	−1.0
SLC25A5	AAC2, ANT 2, T2 (XM_549215)	mitochondrial carrier	* −1.6	1.1	−1.2	−1.2
SLC25A12	AGC1, aralar1 (XM_535962)	mitochondrial carrier	* −1.5	−1.1	−1.2	−1.1
SLC25A37	(XM_543241)	mitochondrial carrier	* −1.7	1.3	−1.0	−1.1
SLC26A11	(XM_540473)	multifunctional anion exchanger	* 3.0	−1.3	−1.0	−1.0
SLC29A1	ENT1 (NM_001003367)	facilitative nucleoside transporter	* −1.9	* 1.7	1.0	1.1
SLC35A1	CST (NM_001003058)	nucleoside-sugar transporter	*1.5	−1.3	−1.1	1.0
SLC35A3	UGTrel2 (NM_001003385)	nucleoside-sugar transporter	* 1.6	−1.1	1.1	1.2
SLC35C1	FUCT1 (XM_540762)	nucleoside-sugar transporter	1.3	−1.4	* 1.7	−1.3
SLC37A4	SPX4, G6PT1 (XM_546493)	sugar-phosphate/phosphate exchanger	* 4.5	* −1.6	−1.0	1.0
SLC38A1	SNAT1, ATA1, GlnT, NAT2 (XM_534827)	System A & N, sodium-coupled neutral amino acid transporter	* −2.6	1.4	−1.1	−1.1

* Indicates statistically significant change of 1.5-fold or higher. Negative ratios indicate decreased expression; Detected and statistically different (18): See table above; Detected but not statistically different (17): SLC-2A8, 6A6, 15A4, 25A4, 25A6, 25A26, 25A36, 26A6, 35B1, 35E2, 38A2, 39A6, 39A10, 39A13, 40A1, 41A2, and 43A2; Not detected (46): SLC-1A2, 1A3, 1A6, 2A3, 2A9, 3A1, 4A4, 4A8, 5A3, 5A10, 5A12, 6A18, 8A1, 9A1, 9A6, 9A9, 10A2, 12A3, 12A6, 12A8, 13A2, 15A1 (PEPT1), 16A4, 17A3, 17A7, 22A1 (OCT1), 22A3 (OCT3), 22A6 (OAT1), 22A9, 22A16, 22A17, 23A2, 24A2, 26A8,C27A5, 30A2, 30A3, 35A2, 35F1, 38A3, 39A12, 41A3, 43A3, SLCO1A2 (OATP-A), SLCO1B3 (OATP8), and SLCO5A1.

**Figure 3:**
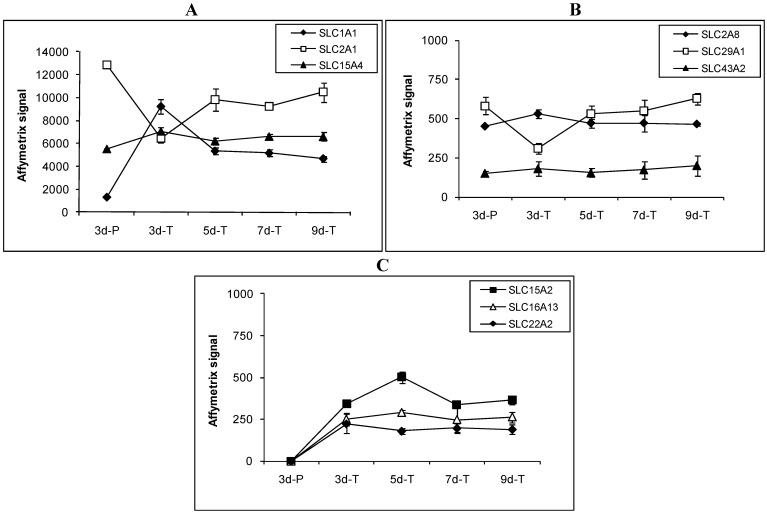
Expression profiles of select SLC transporters involved in drug and nutrient absorption in MDCK cells grown under various conditions. Transporters are divided into 3 groups according to their expression patterns. (**A**) transporters whose Affymetrix signals were above 1000; (**B**) transporters whose Affymetrix signals were below 1000 and expressed in all conditions; and (**C**) transporters whose Affymetrix signals were below 1000 and not expressed on plastic surface.

Among the 36 expressed SLC transporter genes, expression patterns of the genes that are involved in drug and nutrient absorption are described in detail below and in Figure 3. Expression of SLC1A1 (EAA3), a member of the high affinity glutamate and neutral amino acid transporter family, was 4–8 fold higher in MDCK cells grown on the Transwell^®^ membranes than in those grown on plastic plates (Figure 3A). Three other members of the same family, SLC1A2, SLC1A3 and SLC1A6, were not detected. Facilitative GLUT transporters, SLC2A1 (GLUT1) and SLC2A8 (GLUT8), were both detected while SLC2A3 and SLC2A9 were not detected. Expression of SLC2A1 (GLUT1) was higher in cells grown on plastic than on the Transwell^®^ membranes (Figure 3A). SLC2A8 (GLUT8) expression did not change significantly under various conditions (Figure 3B). The apical sodium-dependent bile acid transporter (SLC10A2, ASBT) was not detected in MDCK cells under the conditions studied. 

Proton coupled oligopeptide transporters, SLC15A2 (PEPT2) and SLC15A4 (PHT1), were detected in MDCK cells. SLC15A2 (PEPT2) was expressed at low levels at all times in cells grown on the Transwell^®^ membranes (Figure 3C), but was not detected in MDCK cells grown on plastic. SLC15A4 (PHT1) was expressed at relatively high levels in cells grown on both plastic and the Transwell^®^ membranes-the expression under various conditions did change statistically (Figure 3A). SLC15A1 (PEPT1) was not detected. The monocarboxylic acid transporter SLC16A13 was not detected in cells grown on plastic, but was expressed at low and steady level in cells grown on the Transwell^®^ membranes (Figure 3C). Another member of the monocarboxylic acid transporter family, SLC16A4, was not detected. 

Members of the organic anion transporter family, SLCO1A2 (OATP-A) and SLCO1B3 (OATP8), were not detected in MDCK cells. The organic cation transporter SLC22A2 (OCT2) was not detected in MDCK cells grown on plastic, but was expressed at low levels in cells grown on the Transwell^®^ membranes (Figure 3C). Other members of this family, SLC22A1 (OCT1), SLC22A3 (OCT3), SLC22A6 (OAT1), SLC22A9, SLC22A16, and SLC22A17, were not detected. Expression of the facilitative nucleoside transporter SLC29A1 was doubled in 3d-P compared to that in 3d-T. In cells grown on the Transwell^®^ membranes, expression of SLC29A1 was lowest in 3d-T and highest in 9d-T (Figure 3B). SLC43A2, a bulky neutral amino acid transporter, was expressed at low but steady levels regardless of the growth conditions (Figure 3B). 

#### 2.2.3. Cytochrome P450 (CYP) Enzyme Expression

**Figure 4:**
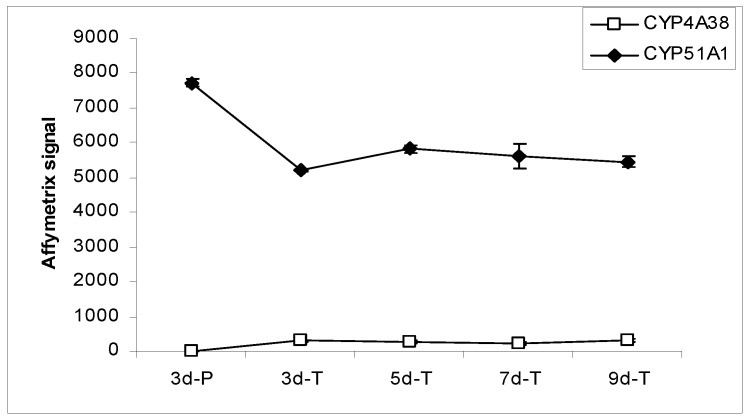
Expression profiles of CYP enzymes in MDCK cells grown under various conditions.

In the Canine GeneChip, CYP genes are represented by 18 probe sets, 14 of which passed the confirmation process. Only 2 CYP genes, CYP4A38 and CYP51A1, were detected in the MDCK cells. CYP4A38 transcripts were not detected in cells grown on plastic, but were detectable at low level in cells grown on the Transwell^®^ membranes (Figure 4). CYP51A1 was expressed at relatively high level in MDCK cells. Its expression on plastic was higher than on the Transwell^®^ membranes while the levels on the Transwell^®^ membranes at various times remained constant. Transcripts of most of the canine CYP genes (*CYP1A2*, *CYP2B11*, *CYP2C21*, *CYP2C41*, *CYP2D15*, *CYP2E1*, *CYP2J2*, *CYP2T1*, *CYP3A12*, *CYP3A26*, *CYP11A1*, and *CYP21A*) represented in the chip were not detected in MDCK cells under the culture conditions studied. 

### 2.3. Transporter Expression in MDCK Cells by qRT-PCR

**Figure 5:**
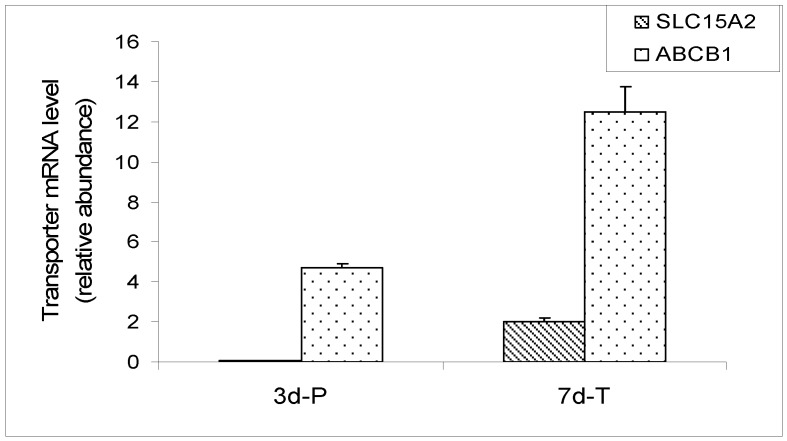
qRT-PCR expression of *SLC15A1* (PEPT1), *SLC15A2* (PEPT2) and ABCB1 (MDR1) in MDCK cells carrying empty vector. The mRNA of PEPT1 was not detected. The data was expressed as relative mRNA abundance after normalization for GAPDH expression.

The microarray data was confirmed by real-time qRT-PCR in a MDCK cell line carrying an empty vector. The cell line displays the same physiological characteristics as the parent MDCK cell line. The mRNA expression of *SLC15A1* (PEPT1), *SLC15A2* (PEPT2) and *ABCB1* (MDR1) transporters was evaluated by qRT-PCR in cells grown on plastic surface for 3 days and on Transwell^®^ membranes for 7 days. The results are expressed as a relative ratio, after normalization with GAPDH expression (Figure 5). Expression of canine PEPT1 was not detectable in the cells grown on either plastic surface or Transwell^®^ membranes. Expression of PEPT2 on plastic was barely detectable, while its expression on Transwell^®^ membranes was readily detectable. The MDR1 expression was easily detectable in MDCK cells at both conditions by RT-PCR. These results are in agreement with the Canine Genechip results, with the exception that PEPT2 expression on plastic was not detected by the array but was marginally detectable by PCR, most likely due to the higher sensitivity of PCR method. 

## 3. Discussion

Affymetrix Canine GeneChip used in this study contained 23,863 unique probe sets representing 16,145 full length genes, but we focused only on the genes that were at least partially annotated in the RefSeq database at the time of data analysis. To further increase the confidence in the expression data, we applied stringent criteria to the probe sets, which resulted in 5554 confirmed probe sets that were present in at least one of the growth conditions (Table 1). Studies from other groups also showed that a large portion of Affymetrix microarray probes (up to 30%–40% depending on the actual chip) were not present in high-quality sequence databases [26,27], indicating the importance of validating the probe sets for the genes of interest in all microarray studies. Though the total number of genes evaluated here accounts for approximately 25% of the dog genome, these are well annotated genes that provide a high confidence in the overall analysis. Real time qRT-PCR of *SLC15A1* (PEPT1), *SLC15A2* (PEPT2) and *ABCB1* (MDR1) genes from cloned MDCK cells under undifferentiated and differentiated conditions confirmed the array results in this study. 

### 3.1. Overall Gene Expression Patterns

Expression of 28% of the genes was statistically different between MDCK cells grown for 3 days on plastic surface or on the Transwell^®^ membranes (Table 2). Genes that exhibited largest difference in the expression between the cells grown for 3 days on plastic and on the Transwell^®^ membranes are involved in a variety of cellular functions including: cell proliferation, migration, differentiation, apoptosis, metabolism and catabolism, signal transduction, transcription, transport, and immune response. However, no apparent patterns for specific biological functions were observed. The dramatic difference in global gene expression profiles is most likely due to the growth conditions. MDCK cells grown on plastic are exposed to media only on the apical surface, while both apical and basolateral layers are exposed to media when cells are grown on the Transwell^®^ membranes. Further, the onset of differentiation and duration of proliferation between cells grown on plastic surfaces and Transwell^®^ membranes are different [24]. Previous studies have suggested that MDCK cells grown on Transwell^®^ membranes attained higher density and differed in the extent of differentiation, cellular morphology, and protein synthesis pattern than the cells grown on regular plastic surfaces [24,25]. The enormous expression difference between cells under the two growth conditions, combined with the lack of obvious patterns of functionalities among genes whose expression changed the most between the two conditions, suggest that they represent distinct cellular states. Therefore, caution should be exercised when interpreting expression and functional data between MDCK cells grown on plastic and the Transwell^®^ membranes. 

Examination of the expression profiles among cells grown on the Transwell^®^ membranes indicated there were significant differences between cells grown for 3 and 5 days. Although there was no statistical enrichment of genes between the MDCK cells grown on the Transwell^®^ membranes for 3 and 5 days, proliferation and/or differentiation related genes were present among the genes whose expression changed the most. For example, six (*G1P2*, *S100A2*, *FABP3*, *FOS*, *TIMP1*, *CTGF*) out of the “top 10” up-regulated genes and four (*IDO*, *AMACO*, *EMP-3*, and *FYN*) out of the “top 10” turned-on genes between 3d-T and 5d-T have been shown to be involved in cell proliferation and/or differentiation. When MDCK cells were allowed to differentiate further (grown for 5, 7, and 9 days on the Transwell^®^ membranes), fewer changes in gene expression were observed (Figure 1, Table 2). This suggested that MDCK cells approached a fully differentiated stage, and their gene expression patterns were stabilized after 5–7 days of growth on the Transwell^®^ membranes. 

### 3.2. Expression of Drug and Nutrient Absorption Related Genes

A small number of genes encoding SLC transporters, ABC transporters, and CYP enzymes were expressed in MDCK cells. Only 13 out of 21 ABC transporter genes, 36 out of 106 SLC transporter genes, and 2 out of 14 CYP genes, were present in at least one of the growth conditions studied. Moreover, only a few of the ones that were detected gave strong hybridization signals. Examination of expression levels for genes important for drug and nutrient absorption indicated that their expressions stabilized after 5 days of culture on the Transwell^®^ membranes (see Figure 2, Figure 3, Figure 4, Table 3, Table 4). This suggests a minimum of 5 day growth period under similar conditions may be required for transport assays using MDCK cells. 

#### 3.2.1. ABC Transporters

ABC transporters play an important role in normal physiology by protecting tissues from toxic xenobiotics and endogenous metabolites. At present, about 48 ABC transporters have been found in human. Based on sequence similarity, dogs have at least 50 ABC transporters, including two additional ABCs (ABCA15 and ABCA16) compared to human. The functions of these two additional ABCs are not clear at this time. Among the 13 ABC transporters expressed in MDCK cells, five (MDR1, MDR3, MRP2, MRP3, and MRP5) are known to be involved in drug and nutrient absorption (Figure 2). MDR1, MDR3 (not in Caco-2), MRP3, and MRP5 were also detected in human intestinal tissues and Caco-2 cells [2,23,28]. Several well studied ABC transporters, such as BSEP, MRP1, and BCRP were not detected in MDCK cells. Previously, others using various detection techniques and functional assays showed that MDCK cells express MRP1 in addition to MDR1 and MRP2 [4,20,23,29,30,31,32,33,34]. The differences observed in MRP1 expression might be due to the more sensitive RT-PCR detection technique and assays employed in their study. The physiological functions of the 8 other transporters (ABCA5, ABCB2, ABCB3, ABCB6, ABCD3, ABCD4, ABCF1, and ABCF2) whose expressions were detected in MDCK cells are not known at this time. To our knowledge, presence of these ABCs in MDCK cells has not been reported previously. 

The expression data suggests that MDCK cells like Caco-2 and human intestinal tissues, express a variety of ABC transporters. MDCK cells transfected with ABC transporters of interest are commonly used to study ABC-mediated drug transport. The presence of the endogenous ABC transporters in MDCK cells that might have different transport kinetics and functional activities as compared to human ABCs could complicate the data interpretation [35].

#### 3.2.2. SLC Transporters

Solute Carrier (SLC) super family is a series of protein transporters that are vital for normal body function by providing nutrients and establishing electrochemical gradients across membranes. To date, about 360 genes that belong to 47 SLC families have been identified in human [36]. SLC transporters are classified based on the type of substrates they transport, but transporters within the same family may exhibit substrate overlap. In MDCK cells, 36 out of the 106 confirmed genes that represent SLC transporter families were detected. Expression patterns of SLC transporters were similar in cells grown on Transwell^®^ membranes for 5 to 9 days, and were very different from cells grown on Transwell^®^ membranes and plastic for 3 days (Table 4). While most of the SLC transporters are involved in providing nutrients, SLCs within SLC10, SLC15, SLC16, SLC22, SLC28, SLC29, SLC47, and SLCO families are particularly important for drug absorption. A number of SLC transporters involved in drug and nutrient absorption were expressed in MDCK cells. 

MDCK cells express a variety of amino acid transporters: SLC1A1, SLC38A1, SLC38A2, and SLC43A2. These results are corroborated by the functional studies that suggested that MDCK cells have low level transport activity for a number of amino acids [31,32,37]. A number of amino acid transporters are also expressed in Caco-2 and human intestine [2,28]. Sodium/bile acid co-transporter family member 2 (SLC10A2) was not detected in MDCK cells. This observation is consistent with the previous reports that MDCK cells do not express bile acid transporters, by either western blot [19] or functional assay [31]. SLC10A2 was detected in human intestine and in differentiated Caco-2 cells but not in undifferentiated Caco-2 cells [2,28]. The high affinity, low capacity proton coupled oligopeptide transporter SLC15A2 (PEPT2) has been well characterized in renal tissues, and its detection in MDCK cells is not surprising [38,39,40,41]. In-contrast, PEPT2 expression was undetectable in both Caco-2 cells and human duodenum. SLC15A1 (PEPT1) was not detected in MDCK cells. PEPT1 was detected at low levels in Caco-2 cells, but at much higher levels in human duodenum [2]. Similar to Caco-2 and human duodenum, SLC15A4 (PHT1), a histidine and selective di- and tri-peptide transporter, exhibited very high hybridization signals in MDCK cells. Previously, SLC15A4 has been reported to be mainly expressed in human skeletal muscle, followed by kidney, heart, and liver, with relatively low expression in colon and brain by northern blot analysis [41,42,43]. This is the first time that presence of PHT1 has been reported in MDCK cells. Terada, *et al*. functionally characterized basolateral peptide transport in MDCK cells and concluded that the renal basolateral peptide transporter was distinguishable from the known peptide transporters, PEPT1 and PEPT2 [44]. Presence of PHT1 in MDCK cells raises the possibility of involvement of this transporter in uptake of dipeptides from basolateral membranes.

The organic anion transporter family (SLC21/SLCO) is one of the largest families of membrane transporters. Members of this family are expressed in many tissues where they are involved in transport of various anionic drugs and nutrients. Interestingly, no OATPs were detected in MDCK cells. Goh, *et al*. also reported absence of OATP-A as well as OATP-C in MDCK cells [23]. Other researchers indicated that MDCK cells functionally express OATP-like genes based on the transport of sulfated substrates [45]. Further expression study is needed to identify this OATP-like gene in MDCK cells. Zalups, *et al*. evaluated OAT1 activity in MDCK cells using organic anion model compound Para-aminohippuric acid (PAH), and the insignificant uptake of PAH indicated OAT1 transporter was not expressed in MDCK cells [46], which is consistent with our findings. SLC22A2 (OCT2) is a polyspecific organic cation transporter critical for elimination of many endogenous small organic cations, as well as a wide array of drugs and environmental toxins in the liver, kidney, intestine, and other organs [47]. In MDCK cells, only OCT2 expression was detected among the several members of this family for which valid probe sets were available. Shu, *et al*. characterized the OCT activity in MDCK cells by functional uptake of organic cation model compound tetraethylammonium (TEA). Only mRNA of OCT2 but not OCT1 and OCT3 was detected, suggesting the OCT activity was mainly due to OCT2 [48], which confirmed our findings. 

There are two types of nucleoside transporters, sodium-dependent concentrative nucleoside transporters (SLC28, CNTs) and sodium-independent equilibrative nucleoside transporters (SLC29, ENTs). Members of these transporter families exhibit some degree of selectivity towards transport of pyrimidine and purine containing nucleoside drugs. In MDCK cells, expression of SLC29A1 (ENT1) was detected. This finding is consistent with the work of Hammond, *et al*., who cloned and functionally characterized this transporter in MDCK cells [49]. Transporters of SLC28 family were not represented in the canine GeneChip. Functionally, MDCK cell monolayers exhibit transport activity for monocarboxylic acid transporter substrates such as benzoic acid [31] and lactic acid [50]. Our results suggest that this function may be performed by SLC16A13, which was detected in MDCK cells.

### 3.3. Cytochrome P450 Enzymes

Cytochromes P450 (CYPs) are a superfamily of enzymes involved in the metabolism of xenobiotics in humans. Currently, 57 CYP genes have been identified in humans, including 15 genes from the CYP 1–3 families which are the major enzymes involved in drug metabolism. Dogs have 51 CYP genes. Based on sequence similarity to the human CYP 1–3 genes, 13 genes in dog are most likely involved in drug metabolism. They are *CYP1A1*, *1A2*, *2A13*, *2A25*, *2B11*, *2C21*, *2C41*, *2D15*, *2E1*, *2E2*, *2F1*, *3A12*, and *3A26*. Among them, 8 genes (*CYP1A2*, *2B11*, *2C21*, *2C41*, *2D15*, *2E1*, *3A12*, and *3A26*) with confirmed probe sets were represented in the canine GeneChip. Our study indicated none of the drug metabolism enzymes listed above was expressed in MDCK cells. Only 2 CYP enzyme genes, *CYP4A38* and *CYP51A1*, were detected in MDCK cells (Figure 4). The CYP51 family is one of the most conserved gene families within the cytochrome P450 super family [51]. *CYP51A1* encodes sterol 14α-demethylase, which catalyzes the oxidative removal of the 14α-methyl group of 14-methyl sterols. *CYP4A38* is the ortholog of human *CYP4A11* and *CYP4A22* genes. CYP4A enzymes function as fatty acid hydroxylases selective for the ω-hydroxylation of saturated and unsaturated fatty acids. To our knowledge, CYP-based metabolism of compounds has not been well characterized in MDCK cells. Lack of expression of CYP enzymes important for drug metabolism suggests that significant CYP mediated metabolism is not expected for most drug compounds in the MDCK *in vitro* model. Previous report suggested that CYP enzymes are expressed at much higher levels in human intestine than Caco-2 cells [2].

## 4. Experimental Section

### 4.1. Tissue Culture

MDCK type II cells (TEER values of ~200 Ω cm^2^) were purchased from ATCC (Manassas, VA). The cells were maintained in Dulbecco’s minimum essential media supplemented with 2 mM L-glutamine, 0.1 mM non-essential amino acids, and 10% FBS at 37 °C in 5% CO_2_ in a humidified incubator. All cell culture reagents were purchased from Mediatech, Inc (Herndon, VA). MDCK cells passage number 62–64 were seeded (1.6 × 10^4^ cells/250 µL/well) in 24-well polycarbonate Transwell^®^ plates (6.5 mm diameter, 0.4 µm pore size, Corning Inc., Corning, NY) coated with collagen type I from rat tail (BD Biosciences, San Diego, CA) and grown for 3, 5, 7, and 9 days. For controls, MDCK cells were seeded at the same density on regular 6-well plastic plates coated with collagen and grown for 3 days. Triplicate samples were prepared for each condition studied. Media was changed every other day. For RT-PCR experiments, cloned MDCK cells were grown for 3 days on plastic and 7 days on Transwell^®^ membranes under the same conditions as described above. 

### 4.2. Microarray Hybridization

Total RNA was isolated from MDCK cells (triplicate samples for each condition) using RNeasy kit from Qiagen (Valencia, CA). The quality of RNA was assessed using an Agilent Bioanalyzer 2100 to compare the ratio of peak heights and area of 28S, 18S, and 5.8S ribosomal RNAs. The RIN values for all the RNA samples used in the microarray analysis were greater than 9.5. For each sample, 2 µg of DNase treated total RNA was converted into double-strand cDNA with an oligo-dT primer containing the T7 RNA polymerase promoter using a cDNA Synthesis System kit from Invitrogen (Carlsbad, CA, USA). In an *in vitro* transcription reaction, biotin-labeled cRNA was generated from the double stranded cDNA using an amplification reagent kit from Affymetrix (Santa Clara, CA), which was then hybridized on Affymetrix^®^ Canine GeneChip^®^. All cDNA and cRNA target preparation steps were processed on a Caliper GeneChip Array Station from Affymetrix. Microarray Suite 5.0 software was used to process the image data obtained from scanning the hybridized array chips. 

### 4.3. RT-PCR

Cloned MDCK cells were lysed directly in the cell culture plates, and total RNA was isolated using RNeasy Mini kit (Qiagen Biosciences, MD). cDNAs were made by reverse transcription of total RNA with oligo d(T)12–18 primers in the presence of Superscript III (Invitrogen, CA, USA). Gene specific primers for PEPT1 (SLC15A1), PEPT2 (SLC15A2) and MDR1 (ABCB1) yielding about 100–150 base pair PCR products were designed based on dog or human gene sequences identified from public databases. The sequences of primers used in this study are published previously [30]. Real-time qPCR was performed in an iCycler using iQ™ SYBR Green Supermix reagent (Bio-Rad, CA, USA). The relative abundance of mRNA in different cells was calculated by determining the number of cycles necessary to reach detection threshold after normalization with GAPDH expression.

### 4.4. Data Analysis

The Affymetrix Canine GeneChip used in this study contained 23,863 unique probe sets representing 16,145 full length genes. Each probe set consists of 11 probe pairs. In order to focus on genes that were at least partially annotated in the RefSeq database at the time of data analysis, all 11 probes of each probe set were blasted against dog RefSeq sequence database. Probe sets that had at least 50% of their probes (6 out of 11) match the gene sequences were considered confirmed. A total of 9045 probe sets in the Canine GeneChip had match in the RefSeq sequences, of these 7908 probe sets passed the confirmation process. Therefore only 7908 confirmed probe sets out of 23,863 total probe sets present in the Canine GeneChip were used for further data analysis.

Hybridization signals were analyzed using Affymetrix MAS 5.0 algorithm. Probe sets with hybridization signal >100 and signal detection as present (*p* value < 0.05) in all triplicate samples of at least one of the growth condition (time point) were assigned as present. Applying these stringent selection criteria resulted in 5554 probe sets that were confirmed and present. For genes represented by more than one probe set, the probe set that was more specific to the corresponding gene was selected. Since most genes in the current analysis were represented by a single probe set, we used the terms “probe set” and “gene” interchangeably throughout this paper. 

Partek^®^ Discovery Suite™ 6.1 software (St. Charles, MO) was used for statistical analysis of the data. Principal Component Analysis (PCA) was carried out to examine similarity of expression profiles among samples at different stages of the differentiation process. Expression difference between various time points was analyzed by *t*-test. The False Discovery Rate (FDR) of 0.05 and expression difference cutoff of 1.5-fold was used to determine statistical significance of expression differences. Gene functions of the validated probe sets were derived from Gene Ontology (GO), annotations in RefSeq and literature sources.

## 5. Conclusions

This study provides the first systematic evaluation of the global expression profiles of genes in MDCK cells grown under differentiation conditions, with particular focus on solute carrier transporters (SLC), efflux transporters (ABC), and cytochrome P-450 enzymes (CYP). The gene expression profile of MDCK cells is strongly influenced by the type of support they are grown on. Therefore, caution should be used when interpreting data between cells grown under different conditions. In MDCK cells, expression of many of the genes including transporters required at least five days of growth on Transwell^®^ membranes to stabilize. Therefore, for transport studies conducted under conditions used here, MDCK cells should be grown on Transwell^®^ membranes for at least five days. It has been shown that permeability data from MDCK cells is similar to permeability data from Caco-2 cells, for passively absorbed compounds [13]. Caco-2 cells, due to significant difference in gene expression levels in Caco-2 cells and human duodenum, are generally not considered a good model for the compounds that are substrates of active transporters [2]. In MDCK cells differentiated on Transwell^®^ membranes, a relatively small number of genes encoding ABCs, SLCs, and CYPs exhibited strong hybridization signals. This suggests that MDCK cells, while a good model for compounds that are passively absorbed, may not be a good model for compounds that are substrate of active transporters. Low expression of the genes involved in drug absorption and dissociation, coupled with their ability to grow and differentiate as monolayers, rapid growth, and ease of transfection, make MDCK cells very useful recombinant models for transport and drug-transporter interaction studies. This study provides valuable reference information on endogenous gene expression in MDCK cells, which could guide researchers in design of drug-transporter and/or drug-enzyme interaction studies, and improve understanding of the contributions of various transporters and metabolic enzymes in MDCK cells. 

## Supplementary Files

Supplementary File 1 Supplementary (PDF, 85 KB)
