# Supplementary material for: Expression Profile of Drug and Nutrient Absorption Related Genes in Madin-Darby Canine Kidney (MDCK) Cells Grown under Differentiation Conditions
Source: Pharmaceutics. 2012 Jun 18;4(2):314–33. doi: 10.3390/pharmaceutics4020314 (PMC3834914; doi:10.3390/pharmaceutics4020314)
Supplement: Supplementary File 1 — Supplementary (PDF, 85 KB) [file pharmaceutics-04-00314-s001.pdf]

Supporting Information

Table S1. List of confirmed SLC transporters represented in Canine GeneChip.

SLC family	Members detected	Members not detected
SLC1: The high affinity glutamate and neutral amino acid transporter family	SLC1A1	SLC1A2, SLC1A3, SLC1A6
SLC2: The facilitative GLUT transporter family	SLC2A1, SLC2A8	SLC2A3, SLC2A9
SLC3: The heavy subunits of the heteromeric amino acid transporters		SLC3A1
SLC4: The bicarbonate transporter family	SLC4A11	SLC4A4, SLC4A8
SLC5: The sodium glucose cotransporter family	SLC5A6	SLC5A3, SLC5A10, SLC5A12
SLC6: The sodium- and chloride- dependent neurotransmitter transporter family	SLC6A6, SLC6A12	SLCA18
SLC7: The cationic amino acid transporter/glycoprotein-associated family	NR	
SLC8: The Na^+/Ca^{2+} exchanger family		SLC8A1
SLC9: The Na^+/H^+ exchanger family		SLC9A1, SLC9A6, SLC9A9
SLC10: The sodium bile salt cotransport family		SLC10A2
SLC11: The proton coupled metal ion transporter family	NR	
SLC12: The electroneutral cation-Cl cotransporter family		SLC12A3, SLC12A6, SLC12A8
SLC13: The human Na^+-sulfate/carboxylate cotransporter family		SLC13A2
SLC14: The urea transporter family	NR	
SLC15: The proton oligopeptide cotransporter family	SLC15A2, SLC15A4	SLC15A1
SLC16: The monocarboxylate transporter family	SLC16A13	SLC16A4
SLC17: The vesicular glutamate transporter family		SLC17A3, SLC17A7
SLC18: The vesicular amine transporter family	NR	
SLC19: The folate/thiamine transporter family	NR	
SLC20: The type III Na^+-phosphate cotransporter family	NR	
SLC21/SLCO: The organic anion transporting family		SLC21A3, SLC21A8, SLC21A15
SLC22: The organic cation/anion/zwitterion transporter family	SLC22A2	SLC22A1, SLC22A3, SLC22A6, SLC22A9, SLC22A16, SLC22A17
SLC23: The Na^+-dependent ascorbic acid transporter family		SLC23A2
SLC24: The $Na^+/(Ca^{2+}$-$K^+)$ exchanger family		SLC24A2
SLC25: The mitochondrial carrier family	SLC25A4, SLC25A5, SLC25A6, SLC25A12, SLC25A26, SLC25A36, SLC25A37	
SLC26: The multifunctional anion exchanger family	SLC26A6, SLC26A11	SLC26A8
SLC27: The fatty acid transport protein family		SLC27A5
SLC28: The Na^+-coupled nucleoside transport family	NR	
SLC29: The facilitative nucleoside transporter family	SLC29A1	
SLC30: The zinc efflux family		SLC30A2, SLC30A3
SLC31: The copper transporter family	NR	

Table S1. *Cont.*

SLC family	Members detected	Members not detected
SLC32: The vesicular inhibitory amino acid transporter family	NR	
SLC33: The Acety-CoA transporter family	NR	
SLC34: The type II Na^+-phosphate cotransporter family	NR	
SLC35: The nucleoside-sugar transporter family	SLC35A1, SLC35A3, SLC35B1, SLC35C1, SLC35E2	SLC35A2, SLC35F1
SLC36: The proton-coupled amino acid transporter family	NR	
SLC37: The sugar-phosphate/phosphate exchanger family	SLC37A4	
SLC38: The System A & N, sodium-coupled neutral amino acid transporter family	SLC38A1, SLC38A2	SLC38A3
SLC39: The metal ion transporter family	SLC39A6, SLC39A10, SLC39A13	SLC39A12
SLC40: The basolateral iron transporter family	SLC40A1	
SLC41: The MgtE-like magnesium transporter family	SLC41A2	SLC41A3
SLC42: The Rh ammonium transporter family (pending)	NR	
SLC43: Na^+-independent, system-L like amino acid transporter family	SLC43A2	SLC43A3
SLC44: Choline-like transporter family	NR	
SLC45: Putative sugar transporter family	NR	
SLC46: Heme transporter family	NR	
SLC47: Multidrug and toxin extrusion family	NR	

NR: confirmed probesets not represented on the Canine GeneChip.

Table S2. Top genes whose expression changed significantly from 3 to 5 days in cells grown on transwells. There were only 6 genes whose expression decreased for more than 2-fold from 3 to 5 days, and only 7 genes whose expression were turned-off from 3 to 5 days.

Gene symbol	RefSeq ID	Gene Name	Function	5d-T/3d-T
		**Top 10 genes up-regulated**		
OAS2	XM_848678	2',5'-oligoadenylate synthetase 2 isoform p69	metabolism	4.1
G1P2	XM_536714	interferon alpha-inducible protein	proliferation	3.8
S100A2	XM_850065	S100 calcium-binding protein A2	cell cycle progression and differentiation	3.1
EPSTI1	XM_534132	epithelial stromal interaction 1	unknown	3
FABP3	XM_535331	Fatty acid-binding protein, heart (H-FABP)	negative regulation of cell proliferation	3
FOS	XM_547914	Proto-oncogene protein c-fos	Transcription factor that regulates proliferation, differentiation, and transformation	2.9
TIMP1	NM_001003182	Tissue inhibitor of metallopeptidase-1	promote cell proliferation, inhibitors of MMPs	2.9
FST	XM_536475	Follistatin precursor (FS) (Activin-binding protein)	negative regulation of FSH secretion	2.8

Table S2. *Cont.*

Gene symbol	RefSeq ID	Gene Name	Function	5d-T/3d-T
CTGF	XM_533406	connective tissue growth factor	adhesion, apoptosis, differentiation, proliferation	2.8
PSMB8	XM_532100	Proteasome subunit beta type 8 precursor (Proteasome component C13)	proteolysis; ubiquitin-dependent protein catabolism; immune response	2.7
		**Top 6 genes down-regulated**		
Vav3 oncogene	XM_537047	Vav-3 protein	intracellular signaling cascade	-2.4
FAM36A	XM_537221	family with sequence similarity 36, member A	unknown	-2.1
SULT1A2	NM_001003223	sulfotransferase family 1A member 2	metabolism	-2.1
STEAP1	XM_532454	Six transmembrane epithelial antigen of prostate 1	channel or pore class transporter	-2.1
RDM1	XM_537910	RAD52 Motif 1	DNA binding and repair	-2.1
PDGFRL	XM_843577	platelet-derived growth factor receptor-like protein	tumor suppressor	-2
		**Top 10 genes turned-on**		
ALDOB	XM_532017	Fructose-bisphosphate aldolase B	metabolism	
CCL17	NM_001003051	chemokine (C-C motif) ligand 17	signaling	
IDO	XM_532793	Indoleamine 2,3-dioxygenase	anti-proliferate	
AMACO	XM_535021	A-domain containing protein similar to matrilin and collagen	differentiation	
B4GALT1	XM_538701	UDP-Gal:β-GlcNAc β-1,4-galactosyltransferase 1	metabolism	
CSF2	NM_001003245	colony stimulating factor 2 (granulocyte-macrophage)	cellular defense response; signal transduction	
transgelin	XM_536561	transgelin	actin binding	
EMP-3	XM_541523	Epithelial membrane protein-3	proliferation	
HMIC	XM_535351	mannosyl-oligosaccharide 1,2-α-mannosidase IC	metabolism	
FYN oncogene	XM_532267	protein-tyrosine kinase fyn isoform c	signal transduction, proliferation, apoptosis	
		**Top 7 genes turned-off**		
ADH4	XM_535665	alcohol dehydrogenase class II pi chain precursor	metabolism	
SAMD11	XM_536715	sterile α motif domain containing 11	unknown	
CHFR	XM_534631	checkpoint with forkhead and ring finger domains	cell cycle	
ENTPD5	XM_537507	ectonucleoside triphosphate diphosphohydrolase 5 precursor	extracellular nucleotides catabolism	
PCDH9	XM_534142	protocadherin 9 isoform 1 precursor	cell adhesion	
EGF	NM_001003094	epidermal growth factor	cell proliferation, etc.	
C6ORF71	XM_533449	chromosome 6 open reading frame 71	unknown	
